# Comprehensive Characterization of the Attenuated Double Auxotroph *Mycobacterium tuberculosis*Δ*leuD*Δ*panCD* as an Alternative to H37Rv

**DOI:** 10.3389/fmicb.2019.01922

**Published:** 2019-08-20

**Authors:** Jomien M. Mouton, Tiaan Heunis, Anzaan Dippenaar, James L. Gallant, Léanie Kleynhans, Samantha L. Sampson

**Affiliations:** ^1^Department of Science and Technology/National Research Foundation (DST/NRF) Centre of Excellence for Biomedical Tuberculosis Research, South African Medical Research Council Centre for Tuberculosis Research, Division of Molecular Biology and Human Genetics, Faculty of Medicine and Health Sciences, Stellenbosch University, Cape Town, South Africa; ^2^Institute for Cell and Molecular Biosciences, Newcastle University, Newcastle upon Tyne, United Kingdom; ^3^Section of Molecular Microbiology, Amsterdam Institute of Molecules, Medicines, and Systems, Vrije Universiteit Amsterdam, Amsterdam, Netherlands

**Keywords:** *Mycobacterium tuberculosis*, biosafety level 2, attenuated auxotroph, model organism, H37Rv

## Abstract

Although currently available model organisms such as *Mycobacterium smegmatis* and *Mycobacterium bovis* Bacillus Calmette-Guérin (BCG) have significantly contributed to our understanding of tuberculosis (TB) biology, these models have limitations such as differences in genome size, growth rates and virulence. However, attenuated *Mycobacterium tuberculosis* strains may provide more representative, safer models to study *M. tuberculosis* biology. For example, the *M. tuberculosis* Δ*leuD*Δ*panCD* double auxotroph, has undergone rigorous *in vitro* and *in vivo* safety testing. Like other auxotrophic strains, this has subsequently been approved for use in biosafety level (BSL) 2 facilities. Auxotrophic strains have been assessed as models for drug-resistant *M. tuberculosis* and for studying latent TB. These offer the potential as safe and useful models, but it is important to understand how well these recapitulate salient features of non-attenuated *M. tuberculosis.* We therefore performed a comprehensive comparison of *M. tuberculosis* H37Rv and *M. tuberculosis*Δ*leuD*Δ*panCD*. These strains demonstrated similar *in vitro* and intra-macrophage replication rates, similar responses to anti-TB agents and whole genome sequence conservation. Shotgun proteomics analysis suggested that *M. tuberculosis*Δ*leuD*Δ*panCD* has a heightened stress response that leads to reduced bacterial replication during exposure to acid stress, which has been verified using a dual-fluorescent replication reporter assay. Importantly, infection of human peripheral blood mononuclear cells with the 2 strains elicited comparable cytokine production, demonstrating the suitability of *M. tuberculosis*Δ*leuD*Δ*panCD* for immunological assays. We provide comprehensive evidence to support the judicious use of *M. tuberculosis*Δ*leuD*Δ*panCD* as a safe and suitable model organism for *M. tuberculosis* research, without the need for a BSL3 facility.

## Introduction

In order to radically reduce TB deaths and incidence by 2030, as set out by the End TB Strategy (World Health Organisation [WHO], 2018a), there is a need for improved TB therapies and more effective ways of studying the deadly pathogen *Mycobacterium tuberculosis*. Current research challenges include restricted access to Biosafety level 3 (BSL3) facilities and the slow growth of *M. tuberculosis*. This emphasizes the need for mycobacterial model systems that can facilitate our understanding of *M. tuberculosis* pathogenesis. Despite studies showing the use of currently available model organisms such as *Mycobacterium smegmatis* and *Mycobacterium bovis* Bacillus Calmette-Guérin (BCG) to have significantly contributed to the understanding of *M. tuberculosis*, these models have limitations.

Apart from being non-pathogenic, *M. smegmatis* is also substantially different from *M. tuberculosis* in terms of its larger genome size and considerably shorter doubling time (Gill et al., 2009; Mouton et al., 2016). The model organism BCG is known to contain a natural RD1 deletion (Behr et al., 1999), which encodes the known virulence factors early secreted antigenic target-6 kDa (ESAT-6) (Harboe et al., 1996) and culture filtrate protein-10 kDa (CFP-10) (Lewis et al., 2003; Gao et al., 2004; Guinn et al., 2004; Fortune et al., 2005), suggesting that the immune response elicited by BCG will be altered in comparison to *M. tuberculosis*.

Several attenuated strains of *M. tuberculosis* have been developed (Hondalus et al., 2000; Sambandamurthy et al., 2002, 2005; Sampson et al., 2004, 2011; Derrick et al., 2007; Clemmensen et al., 2017; Kar et al., 2017; Bahal et al., 2018) that have the potential to serve as model organisms to study *M. tuberculosis* biology. These include strains with a deletion in the *bioA* gene, disrupting biotin synthesis (Kar et al., 2017) or single mutation in the *eccCa1* gene, disrupting ESX-1 type VII secretion, resulting in reduced host immune responses and immunopathology (Clemmensen et al., 2017). To improve safety, several *M. tuberculosis* strains with two or more attenuating mutations have been developed. These include Δ*RD1*Δ*panCD* (Sambandamurthy et al., 2002, 2005), which similarly to BCG does not include the RD1 region. Doubly auxotrophic strains include Δ*lysA*Δ*panCD* (Sambandamurthy et al., 2002, 2005) and Δ*leuD*Δ*panCD* (Sampson et al., 2004). Recent work generated a drug-susceptible and drug-resistant triple auxotrophic stain of *M. tuberculosis*, which provides a safe model for studying drug-resistant *M. tuberculosis* under BSL2 conditions (Vilchèze et al., 2018). Another interesting development is the use of a streptomycin-dependent *M. tuberculosis* strain (*M. tuberculosis* 18b) as a model of latent TB (Zhang et al., 2012). These strains offer potential as research models, but need comprehensive characterization to assess their suitability to the research question at hand.

The severely attenuated double auxotrophic Δ*leuD*Δ*panCD* strain of *M. tuberculosis* (Sampson et al., 2004, 2011) offers significant advantages over *M. smegmatis* and BCG such as similar genetic background, growth and antigenicity properties to the widely used laboratory strain *M. tuberculosis* H37Rv. For example, the growth rate of the Δ*leuD*Δ*panCD* strain in minimal medium supplemented with both leucine and pantothenate was shown to be similar to that of wild-type *M. tuberculosis* in minimal medium (Sampson et al., 2004). Importantly, although the strain is fully attenuated, it was shown to retain immunogenicity and protective capacity in a sensitive guinea pig TB aerosol challenge model (Sampson et al., 2004, 2011). In addition, the severely attenuated mutant of *M. tuberculosis* has undergone rigorous *in vitro* and *in vivo* safety testing, since it was originally developed as a TB vaccine candidate (Sampson et al., 2004, 2011). The Δ*leuD*Δ*panCD* strain proved to be highly attenuated in the severe combined immune deficient mouse *M. tuberculosis* model (Sampson et al., 2004). The safety of this strain was further supported by evidence that it does not cause disease in simian immunodeficiency virus (SIV)-co-infected Rhesus macaques (Sampson et al., 2011). Collectively this data indicates that in the case of accidental infection with this strain in humans, it would be highly unlikely to cause disease, even in immune-compromised hosts. The Δ*leuD*Δ*panCD* strain, therefore, holds minimal risk to human health and environment and serves as an excellent alternative model organism for TB research. Currently, several international laboratories have been granted approval to work with this and similar strains, under BSL2 conditions (Movahedzadeh et al., 2008; Vilchèze et al., 2018).

We therefore aimed to compare *M. tuberculosis* H37Rv and *M. tuberculosis* Δ*leuD*Δ*panCD* to assess the suitability of this auxotrophic strain as a model for *M. tuberculosis* research. We provide comprehensive comparative analyses between *M. tuberculosis* H37Rv and *M. tuberculosis*Δ*leuD*Δ*panCD* with regards to *in vitro* and intra-macrophage growth, genomic background, response to anti-TB agents, proteomic response to stress, and the host immune response.

## Materials and Methods

### Bacterial Strains and Culture

All bacterial strains utilized in this study are listed and described in Table 1, along with relevant plasmid information. All reagents were purchased from Sigma-Aldrich unless otherwise specified. Liquid cultures of mycobacterial strains were grown in Middlebrook 7H9 supplemented with 10% oleic acid-albumin-dextrose-catalase (OADC, Becton Dickinson, NJ, United States), 0.2% (v/v) glycerol and 0.05% (v/v) Tween 80 (7H9-OGT), with antibiotics as required for plasmid maintenance, at 37°C, with shaking at 180 rpm. *M. tuberculosis*Δ*leuD*Δ*panCD* liquid cultures were additionally supplemented with 50 μg/ml leucine and 24 μg/ml pantothenate. Electro-competent mycobacteria were prepared and transformed as described by Snapper et al. (1990). Solid media cultures of mycobacteria were grown on 7H10 agar supplemented with 10% OADC, 0.5% (v/v) glycerol and antibiotics at 37°C and in the case of *M. tuberculosis*Δ*leuD*Δ*panCD*, 50 μg/ml leucine and 24 μg/ml pantothenate. Mycobacterial strains expressing the bacterial luciferase operon from plasmid pMV306hsp + LuxCDABE (Andreu et al., 2010) do not require an exogenous substrate to produce light. Bioluminescence was used to measure the intracellular growth of both the reference strain *M. tuberculosis* H37Rv and *M. tuberculosis*Δ*leuD*Δ*panCD.* The number of generations were calculated based on either OD, luminescence or median fluorescence intensity as previously described (Mouton et al., 2016).

**TABLE 1:** Plasmids and strains.

**Plasmid/strain**	**Description**	**Source**
pTiGc	*hsp60(ribo)-turboFP635 hsp60-gfp*, Kan^*R*^, episomal	Mouton et al. (2016), Addgene plasmid 78314
pMV306hsp + LuxCDABE	Bacterial luciferase operon, Kan^*R*^, episomal	Andreu et al. (2010), Addgene plasmid number 26519
*M. smegmatis* mc^2^155	Non-pathogenic, fast-growing model organism	ATCC 700084
*M. tuberculosis*Δ*leuD*Δ*panCD*	Double leucine and pantothenate auxotroph	Sampson et al. (2004), gift from Prof. Bill Jacobs
*M. tuberculosis* H37Rv	*M. tuberculosis* reference strain	Gift from Prof. Barry Bloom

**ATCC, American Type Culture Collection; Hyg^*R*^, hygromycin resistant; Kan^*R*^, kanamycin resistant.**

For proteomic analysis, *M. tuberculosis* H37Rv and *M. tuberculosis*Δ*leuD*Δ*panCD* were cultured, separately, in Middlebrook 7H9 broth supplemented with dextrose-catalase (DC), 0.2% (v/v) glycerol and 0.05% (v/v) Tween 80 (7H9-DC) at 37°C. Mycobacterial cells were harvested (4000 rpm, 10 min, 4°C) at an OD_600_ of ∼0.8, and the cells were washed twice with ice-cold phosphate-buffered saline (PBS) pH 7.4. Cells were washed with PBS to remove residual media before LC-MS/MS analyses. Cells were either stored at −20°C until further processing (control), or resuspended in 7H9-DC (pH 4.5) and incubated for 48 h at 37°C. Acid-stressed cultures were subsequently washed twice by centrifugation at 4000 rpm for 10 min at 4°C with ice-cold PBS pH7.4, and the pellets were stored at −20°C.

For testing the effect of acid stress on bacterial replication using a dual-fluorescent replication reporter previously developed by our group (Mouton et al., 2016), bacteria were grown in 7H9-DC containing 4 mM Theophylline, to induce the expression of TurboFP635, for 7 days until an OD_600_ of ∼0.8 before washing with PBS. The cultures were sub-cultured in 7H9-DC without Theophylline at pH 6.5 and pH 4.5 and incubated for 48 h at 37°C.

### Genomic DNA Extraction

Genomic DNA was extracted by pelleting 15 ml culture at OD_600_ of 0.8 for 10 min at 4000 rpm and proceeding according to previously published methods (Somerville et al., 2005).

### Whole Genome Sequencing (WGS)

Whole genome sequencing was done on an Illumina NextSeq 550 instrument (Illumina, CA, United States) using a paired-end approach with ∼600 base fragment sizes. One microgram of DNA was used to prepare libraries for sequencing per the manufacturer’s instructions using the NEBNext Ultra DNA library preparation kit for Illumina (New England Biolabs, MA, United States).

### WGS Data Analyses

The Illumina paired-end reads for all isolates were analyzed with open source software as described previously (Black et al., 2015; Dippenaar et al., 2015). Identified variants were compared between *M. tuberculosis* H37Rv and *M. tuberculosis*Δ*leuD*Δ*panCD*. Deleted regions in the genomes with respect to the *M. tuberculosis* H37Rv NC000962.3 reference genome were detected with DELLY and alignments of these regions were visually inspected (Rausch et al., 2012).

### MIC Determination Using BACTEC MGIT 960

Minimum inhibitory concentration (MIC) determinations were performed using the semi-automated liquid culture BACTEC MGIT 960 system (Becton Dickinson) and EpiCenter software equipped with a TB eXist module for drug susceptibility testing (DST) (Springer et al., 2009). Briefly, a bacterial suspension was prepared from MGIT subcultures according to the manufacturer’s instructions (BACTEC^TM^ MGIT^TM^ 960 System User’s Manual: Becton Dickinson Document Number MA-0117) and 0.5 ml of the suspension was added to each MGIT tube supplemented with 0.8 ml of OADC and 0.1 ml of the drug (dissolved in DMSO) at a concentration range of 0.06–9.0 μg/ml for rifampicin or 0.0015–2.0 μg/ml for isoniazid. *M. tuberculosis*Δ*leuD*Δ*panCD* was supplemented with 50 μg/ml leucine and 24 μg/ml pantothenate. The MIC was determined as the lowest drug concentration that tested susceptible (less than 100 growth units by automated reading when the control vial turned positive).

### Mammalian Cell Culture

RAW264.7 cells (ATCC TIB-71) were cultured in Dulbecco’s Modified Eagle’s Medium (DMEM), supplemented with 10% heat-inactivated fetal bovine serum (FBS) at 37°C in 5% CO_2_. Cells were passaged every 2–4 days. For infections, cells were seeded at 5 × 10^4^ cells per well in 96 well white plates. *M. tuberculosis* H37Rv and *M. tuberculosis*Δ*leuD*Δ*panCD* were prepared and infected into RAW264.7 macrophages as described before (Mouton et al., 2016). Bacteria were added to macrophages at a 10:1 ratio, and incubated at 37°C in 5% CO_2_ for 3 h, prior to penicillin/streptomycin treatment and subsequent washing, to remove extracellular bacteria. Infected RAW264.7 macrophages were cultured in the presence of DMEM, supplemented with 10% FBS, 50 μg/ml leucine and 24 μg/ml pantothenate to allow growth of the auxotrophic strain inside macrophages. To assess the uptake of bacteria by macrophages, cells were lysed with sterile distilled water and pipetting, followed by colony forming unit (CFU) determination by serial dilution plating of lysates onto 7H10 agar, with leucine and pantothenate supplementation where necessary. Macrophages infected with strains expressing the bacterial luciferase operon were assessed for bioluminescence expression using a microplate reader (POLARstar Omega, BMG Labtech). Intracellular growth was monitored by measuring bioluminescence every 24 h for 3 days. Macrophages infected with mycobacteria that do not contain the luciferase operon were included as controls to subtract background luminescence expression from all samples.

### Isolation and Infection of Peripheral Blood Mononuclear Cells (PBMCs)

PBMCs were isolated from whole blood, of healthy TST negative donors (*n* = 12), using Ficoll-Paque PLUS (GE Healthcare Life Sciences, MA, United States) density (*D* > 1.077 g/ml) gradient centrifugation. Informed consent was obtained from all the subjects and the study was approved by the Ethical Review Committee of the Faculty of Health Sciences at Stellenbosch University (N16/05/070). Cells were cultured in Roswell Park Memorial Institute (RPMI) media, supplemented with 10% FBS at a density of 5 × 10^5^ cells per well in 48-well plates (Greiner Bio-one, Kremsmünster, Austria). PBMCs were then infected with *M. tuberculosis* H37Rv or *M. tuberculosis*Δ*leuD*Δ*panCD* at an MOI of 10:1, treated with penicillin/streptomycin, followed by washing as described above, before adding fresh RPMI, containing 10% FBS. Uninfected and Lipopolysaccharide- (LPS; 10 μg/ml) stimulated cells were included as negative and positive controls, respectively. Supernatants were collected 24 h post-infection and stored at -80°C until cytokine analysis. To assess uptake, PBMCs were lysed with sterile distilled water and pipetting, followed by serial dilution plating and CFU determination as described above.

### Quantification of Cytokine and Chemokine Levels by Multiplex Bead Array

A human ProcartaPlex^TM^ Multiplex Immunoassay (Thermo Fisher Scientific, MA, United States) was used to simultaneously quantify the levels of the following analytes in the culture supernatants: interleukin (IL)-1β, IL-12p70, granulocyte monocyte stimulating factor (GM-CSF), growth-regulated oncogene (GRO)α, interferon (IFN)γ, macrophage inflammatory protein (MIP)-1α (CCL3), tumor necrosis factor (TNF)α, RANTES, stromal cell-derived factor (SDF)-1α. The assays were performed according to the manufacturer’s instructions and samples were evaluated in triplicate. The cytokine concentrations were measured on a Bio-Plex platform (Bio Plex^TM^, Bio-Rad Laboratories, CA, United States). A standard curve ranging from 227 to 8,979.52 pg/ml for IL-1β, 7.15–29,426.85 pg/ml for IL12-p70, 13.69–51,379.68 pg/ml for GM-CSF, 1.93–9993 pg/ml GROα, 11.67–49,632.42 pg/ml for IFNγ, 1.5–6755.47 pg/ml for MIP-1α, 7.85–33,043.15 pg/ml for TNFα, 0.86–837.85 pg/ml for RANTES and 10.85–34,295.44 pg/ml for SDF-1, was used in the assay. Correlation coefficients (*r*^2^ > 0.9) for the standard curves were determined from transformed mean fluorescent intensity values for each cytokine. Bio-Plex Manager Software, version 6.1, was used to determine the median fluorescent intensities.

### Proteomic Sample Preparation and LC-MS/MS Analysis

Mycobacterial cells from 25 ml culture (performed in four independent replicates) were mechanically lysed and whole-cell lysates were processed for LC-MS/MS analysis using a modified version of the filter-aided sample preparation (FASP) approach (Wiśniewski et al., 2009). A total of 1 μg peptide mixture from each sample was analyzed, independently, on an Orbitrap Fusion Tribrid mass spectrometer (Thermo Fisher Scientific, MA, United States), connected to a Thermo Scientific UltiMate 3000 RSLCnano System (Thermo Fisher Scientific, MA, United States) (Detailed information is provided in Supplementary Data Sheet S1).

### Flow Cytometry Sample Preparation, Acquisition, and Analyses

Samples were sonicated, fixed in 4% formaldehyde for 30 min and washed twice in PBS, containing 0.05% Tween as previously described (Mouton et al., 2016). Samples not immediately analyzed were then stored at 4°C. Immediately prior to flow cytometry analyses samples were pelleted, resuspended in PBS and filtered. Samples were analyzed using a FACSJazz flow cytometer (Beckton Dickinson) for GFP fluorescent intensity using a 488 nm laser (530/40 filter) and TurboFP635 fluorescent intensity using a 561 nm laser (610/20 filter). For each sample, 30,000 events were captured and flow cytometry data were analyzed using FlowJo vX.0.07r2 software. The number of generations were calculated based on fluorescence intensity data as described before (Mouton et al., 2016). Generation times are expressed as mean ± SD.

### Data Analysis

We used an exploratory data analysis approach for the multiplex bead array assay. Details are indicated in Supplementary Data Sheet S1. All tandem mass spectra were analyzed using MaxQuant 1.5.5.1 (Cox and Mann, 2008), and searched against a customized *M. tuberculosis* proteome database. Custom database construction was performed as previously described (Heunis et al., 2017) and are detailed in Supplementary Data Sheet S1. Exploratory data analysis and visualization were performed in the R statistical programming language^1^.

## Results

### Next-Generation Sequencing Reveals Sequence Conservation Between *M. tuberculosis*Δ*leuD*Δ*panCD* and *M. tuberculosis* H37Rv

Whole genome sequencing of *M. tuberculosis*Δ*leuD*Δ*panCD* and *M. tuberculosis* H37Rv (the reference strain in use in our laboratory) was used to confirm sequence conservation outside of the *leuD* and *panCD* regions. When comparing *M. tuberculosis*Δ*leuD*Δ*panCD* to our laboratory reference strain *M. tuberculosis* H37Rv, only one unique non-synonymous variant (I131T A > G) was identified at position 392 in Rv2988 (*leuC*) in *M. tuberculosis*Δ*leuD*Δ*panCD* that was not found in *M. tuberculosis* H37Rv. However, no peptide covering this region was identified in either of the two strains. Importantly, the *leuC* gene is located upstream of the *leuD* deletion and no proteomic differences were observed in downstream proteins in the attenuated and wild type strains, suggesting an absence of polar effects on expression. In agreement with the findings of Ioerger et al. (2010), our analysis identified 33 variants in both the attenuated *M. tuberculosis*Δ*leuD*Δ*panCD* strain and *M. tuberculosis* H37Rv laboratory strain with respect to the reference strain *M. tuberculosis* H37Rv, NC000962.3 (Ioerger et al., 2010). However, 32 of these variants were identical between the attenuated *M. tuberculosis*Δ*leuD*Δ*panCD* and the *M. tuberculosis* H37Rv laboratory strain (Supplementary Table S1). Visual inspection of the alignment confirmed the expected 1297 bp *panCD* locus deletion at position 4043882–4045179 (*M. tuberculosis* H37Rv gene Rv3602c and Rv3601c) and the *leuD* deletion at position 3344036–3344394 (*M. tuberculosis* H37Rv gene Rv2987C) in the attenuated *M. tuberculosis*Δ*leuD*Δ*panCD*, with respect to *M. tuberculosis* H37Rv, NC000962.3. At a genomic level, *M. tuberculosis*Δ*leuD*Δ*panCD* is therefore highly similar to *M. tuberculosis* H37Rv.

### The *in vitro* and Intra-Macrophage Growth of *M. tuberculosis*Δ*leuD*Δ*panCD* and *M. tuberculosis* H37Rv Are Comparable

**FIGURE 1:**
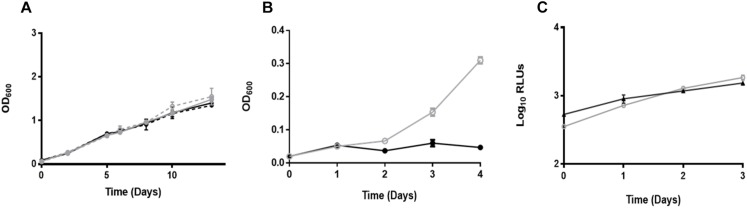
Comparable growth of *M. tuberculosis*Δ*leuD*Δ*panCD* and *M. tuberculosis* H37Rv strains. **(A)** *M. tuberculosis*Δ*leuD*Δ*panCD* (gray solid line, closed symbols), *M. tuberculosis*Δ*leuD*Δ*panCD* containing the dual reporter pTiGc (gray dotted line, open symbols), *M. tuberculosis* H37Rv (black solid line, closed symbols) and *M. tuberculosis* H37Rv containing pTiGc (black dotted line, open symbols) growth was monitored by OD. One-way ANOVA with Tukey’s multiple comparisons test indicated no significant difference (*p* > 0.05). **(B)** OD_600_-based *M. tuberculosis*Δ*leuD*Δ*panCD* growth with exogenous supplementation of leucine and pantothenate (gray line, open symbols) and without pantothenate supplementation (black line, closed symbols). **(C)** RAW264.7 macrophages were infected with *M. tuberculosis*Δ*leuD*Δ*panCD* + LuxCDABE (gray line, open symbols) *or M. tuberculosis* H37Rv + LuxCDABE (black line, closed symbols), and intracellular mycobacterial replication was compared by monitoring bioluminescence. Data shown are depicted as mean ± SD of three technical replicates and are representative of three independent biological replicates. Pearson correlation tests revealed a statistically significantly positive correlation between generations calculated using luminescence for *M. tuberculosis*Δ*leuD*Δ*panCD* and *M. tuberculosis* H37Rv (*r* = 0.975, *N* = 4, *p* = 0.0250).

To confirm that the growth rates of *M. tuberculosis*Δ*leuD*Δ*panCD* and *M. tuberculosis* H37Rv were similar *in vitro*, samples were taken for OD measurements at predetermined time points (Figure 1A). Strains containing the pTiGc plasmid were included to determine whether plasmid carriage affected bacterial growth. One-way ANOVA with Tukey’s multiple comparisons test revealed no differences in growth rates between these strains (*p* > 0.05). To confirm the auxotrophic nature of the *M. tuberculosis*Δ*leuD*Δ*panCD* strain (Sampson et al., 2004), OD measurements taken from strains supplemented exogenously with leucine and pantothenate showed normal growth, whereas restricted growth was observed in the absence of pantothenate (Figure 1B).

We next assessed replication of *M. tuberculosis* during macrophage infection. RAW264.7 macrophages were infected with *M. tuberculosis*Δ*leuD*Δ*panCD* and *M. tuberculosis* H37Rv strains expressing the bacterial luciferase operon. Bioluminescence measurements demonstrated no difference in the growth of these strains, suggesting similar intracellular replication of *M. tuberculosis*Δ*leuD*Δ*panCD* and *M. tuberculosis* H37Rv (Figure 1C). Pearson correlation tests revealed a statistically significantly positive correlation between generations calculated using luminescence for *M. tuberculosis*Δ*leuD*Δ*panCD* and *M. tuberculosis* H37Rv (*r* = 0.975, *N* = 4, *p* = 0.0250).

### *M. tuberculosis*Δ*leuD*Δ*panCD* and *M. tuberculosis* H37Rv Respond Similarly to Anti-tuberculosis Agents

One important potential application of the attenuated *M. tuberculosis*Δ*leuD*Δ*panCD* strain is to allow for anti-mycobacterial compound screening. Phenotypic DST of the two first-line anti-tuberculosis drugs with different mechanisms of action, rifampicin and isoniazid, confirmed similar MICs for *M. tuberculosis*Δ*leuD*Δ*panCD* and *M. tuberculosis* H37Rv. Specifically, the MIC for isoniazid was determined to be 0.06 μg/ml for both strains, and the MIC for rifampicin was determined to be 0.25 μg/ml for *M. tuberculosis*Δ*leuD*Δ*panCD* and 0.5 μg/ml for *M. tuberculosis* H37Rv.

### Proteomic Analysis Reveals an Increased Stress Response in *M. tuberculosis*Δ*leuD*Δ*panCD*

**FIGURE 2:**
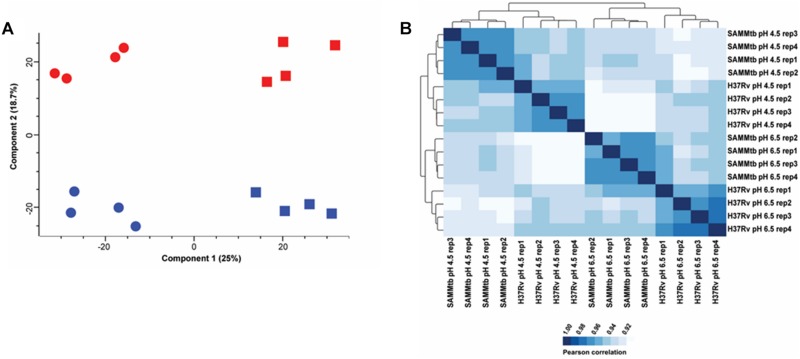
Quality control analysis of proteomics data reveals distinct clustering of *M. tuberculosis* H37Rv and *M. tuberculosis*Δ*leuD*Δ*panCD* protein intensities. **(A)** Principle component analysis of z-scored MaxQuant LFQ intensities obtained from *M. tuberculosis* H37Rv (blue) and *M. tuberculosis*Δ*leuD*Δ*panCD* (red) during control (pH 6.5; square) and acid stress conditions (pH 4.5; circle). **(B)** Correlogram of Pearson correlation coefficients from MaxQuant LFQ intensities obtained for *M. tuberculosis* H37Rv and *M. tuberculosis*Δ*leuD*Δ*panCD* (*SAMMtb*) cultured at pH 6.5 and pH 4.5.

We performed LC-MS/MS analysis on the proteomes of *M. tuberculosis*Δ*leuD*Δ*panCD* and *M. tuberculosis* H37Rv grown in media at pH 6.5 and pH 4.5. In total, we identified 21,779 unique peptides that mapped to 2,329 proteins, which contained ≥ 2 unique peptides at an empirical protein FDR of <1% (Supplementary Tables S2, S3). The proteins identified in this study covered 58.33% of the predicted *M. tuberculosis* H37Rv proteome. Principle component analysis revealed distinct clustering of replicates and experimental groups (Figure 2A). The first principle component (Component 1) explained 25% of the variance in the data, which has an association with acid stress. The second principle component (Component 2) explained ∼19% of the variance in the data and was associated with inherent strain differences between *M. tuberculosis* Δ*leuD*Δ*panCD* and *M. tuberculosis* H37Rv. Additionally, Pearson correlation coefficients were higher within biological replicates than between groups (Figure 2B), indicating high reproducibility between independent experiments. Hierarchical clustering revealed two major clusters that separated acid stressed *M. tuberculosis* Δ*leuD*Δ*panCD* and *M. tuberculosis* H37Rv from the strains grown under control (pH 6.5) conditions. Sub-clusters separated *M. tuberculosis*Δ*leuD*Δ*panCD* and *M. tuberculosis* H37Rv. Taken together this data indicates that exposure to acid stress induced more variance in the data than the inherent strain differences between *M. tuberculosis*Δ*leuD*Δ*panCD* and *M. tuberculosis* H37Rv, with the strains having similar protein expression profiles under the conditions tested. However, some proteome-level differences were observed between the two strains grown under control (pH 6.5) and acid stress (pH 4.5) conditions.

**FIGURE 3:**
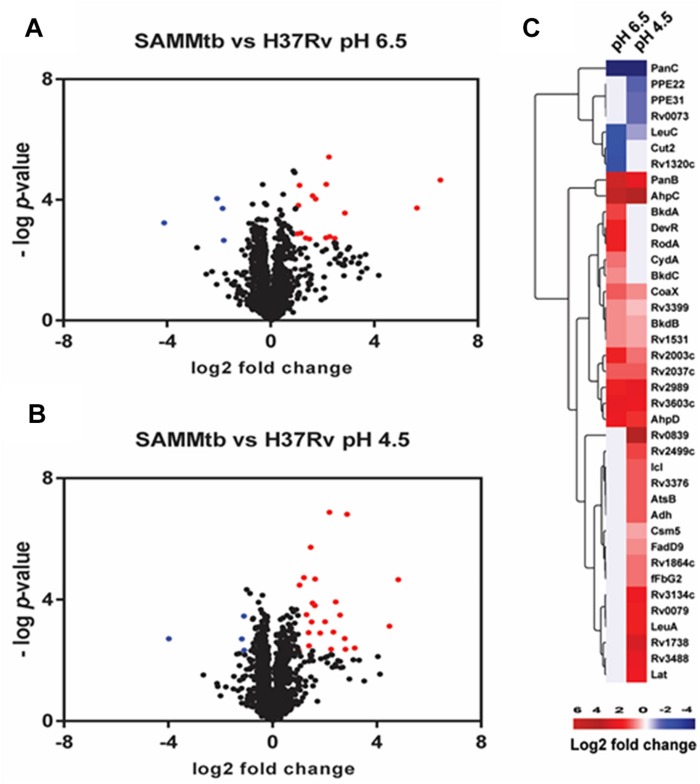
Proteomic comparison of *M. tuberculosis* H37Rv and *M. tuberculosis*Δ*leuD*Δ*panCD* under physiological *in vitro* growth conditions and in response to acid stress. Volcano plot showing protein expression differences between *M. tuberculosis* H37Rv compared to *M. tuberculosis*Δ*leuD*Δ*panCD* when grown at pH 6.5 **(A)** and pH 4.5 **(B)**. Blue corresponds to proteins with <-1 log_2_ fold differential expression and adjusted *p* < 0.05. Red corresponds to proteins with > 1 log_2_ fold differential expression and adjusted *p* < 0.05. **(C)** Heatmap of log_2_ fold changes of proteins showing statistically significant regulation between *M. tuberculosis* H37Rv and *M. tuberculosis*Δ*leuD*Δ*panCD* during growth at pH 6.5 and pH 4.5.

**FIGURE 4:**
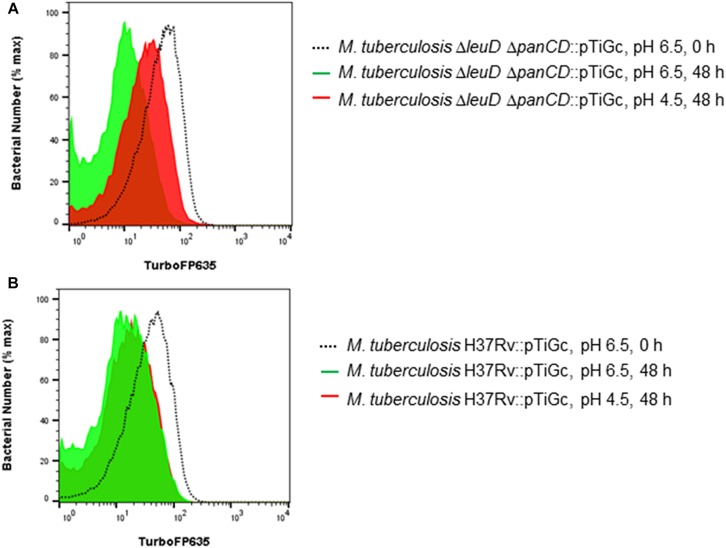
Fluorescence dilution demonstrates reduced replication of *M. tuberculosis*Δ*leuD*Δ*panCD* under acid stress in comparison to *M. tuberculosis* H37Rv. *M. tuberculosis* H37Rv and *M. tuberculosis*Δ*leuD*Δ*panCD* containing pTiGc was cultured in the presence of 4 mM theophylline (Theo), before removal of theophylline and exposure to pH 4.5 and pH 6.5 for 48 h prior to analyses by flow cytometry. Flow cytometry histograms demonstrate increased TurboFP635 fluorescence intensity in acidic media (pH 4.5, red), compared to normal media (pH 6.5, green) in *M. tuberculosis*Δ*leuD*Δ*panCD* **(A)** after 48 h. In contrast, *M. tuberculosis* H37Rv demonstrated similar, reduced, TurboFP635 fluorescence intensity after 48 h in acidic media (pH 4.5, red) and control media (pH 6.5, green) **(B)**. Representative examples of three independent biological repeats are shown.

We performed pair-wise comparisons to identify differences in relative protein abundances between *M. tuberculosis* H37Rv and *M. tuberculosis*Δ*leuD*Δ*panCD*. Twenty differentially regulated proteins, with a twofold change and adjusted *p* < 0.05, were identified when both of these strains were grown at pH 6.5 (Figure 3A and Supplementary Table S4). We detected 28 proteins as differentially regulated in *M. tuberculosis*Δ*leuD*Δ*panCD* during acid stress, compared to *M. tuberculosis* H37Rv (Figure 3B and Supplementary Table S5). Interestingly, we observed 8 proteins that were more abundant in acid-stressed *M. tuberculosis*Δ*leuD*Δ*panCD* cells that were also detected as more abundant in *M. tuberculosis*Δ*leuD*Δ*panCD* grown under physiological conditions, compared to *M. tuberculosis* H37Rv under the same conditions (Figure 3C). These included proteins that play a role in dormancy, oxidative and/or nitrosative stress (AhpC, AhpD), pantothenate metabolism (CoaX, PanB, Rv3603c), transcription (Rv2989), methyl transfer (Rv2003c) and lipid catabolism (Rv2037c). This, in addition to increased abundance of DevR in *M. tuberculosis*Δ*leuD*Δ*panCD* during growth at pH 6.5, may suggest that *M. tuberculosis* Δ*leuD*Δ*panCD* experiences a more pronounced baseline stress response than *M. tuberculosis* H37Rv, which could be exacerbated during exposure to stress and could lead to reduced bacterial replication. To test this hypothesis we exploited a previously described dual-fluorescent replication reporter and flow cytometry to assess the effect of stress on bacterial replication. In accordance with our proteomics analyses, *M. tuberculosis*Δ*leuD*Δ*panCD* demonstrated a more pronounced decrease in bacterial replication in response to acid stress, compared to *M. tuberculosis* H37Rv after 48 h (Figure 4).

### *M. tuberculosis*Δ*leuD*Δ*panCD* Induces Similar or Higher PBMC Cytokine and Chemokine Responses Compared to H37Rv

**FIGURE 5:**
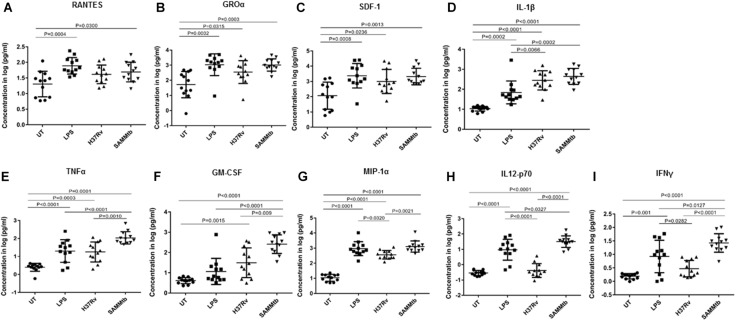
Cytokine secretion in response to *M. tuberculosis*Δ*leuD*Δ*panCD* and *M. tuberculosis* H37Rv. Cytokine release was assessed by Luminex analyses of supernatants from PBMCs. Cells were seeded at 5 × 10^5^ cells per well in a 48 well plate and infected with *M. tuberculosis*Δ*leuD*Δ*panCD* and *M. tuberculosis* H37Rv (MOI 10:1) for 24 h. **(A)** RANTES, **(B)** Groα, **(C)** SDF-1, **(D)** IL-1β, **(E)** TNFα, **(F)** GM-CSF, **(G)** MIP-1α, **(H)** IL12-p70, **(I)** IFNγ. Lipopolysaccharide (LPS, 10 μg/ml)-stimulated and uninfected cells were included as controls. Supernatants were analyzed by multiplex assays using the Bio-Plex platform. The log-transformed data were analyzed using a one-way ANOVA with a Tukey Honest Significant Differences (HSD) *post hoc* test. A *p* < 0.05 was regarded as significantly different. All experiments were performed in technical triplicates.

The concentration of 9 immune markers including cytokines, chemokines and growth factors were measured in the culture supernatant of PBMCs 24 h after infection with *M. tuberculosis*Δ*leuD*Δ*panCD* and *M. tuberculosis* H37Rv. No statistically significant differences were observed in RANTES, GROα, SDF-1, and IL-1β concentrations between *M. tuberculosis*Δ*leuD*Δ*panCD* and *M. tuberculosis* H37Rv (Figures 5A–D). Despite demonstrating similar trends, *M. tuberculosis*Δ*leuD*Δ*panCD* did induce higher concentrations of TNFα (*p* = 0.0010), GM-CSF (*p* = 0.009), MIP-1α (*p* = 0.0021), IL-12p70 (*p* < 0.0001), and IFNγ (*p* < 0.0001) when compared to *M. tuberculosis* H37Rv (Figures 5E–I). *M. tuberculosis* H37Rv induced low concentrations of IL-12p70 and IFNγ. Interestingly, *M. tuberculosis*Δ*leuD*Δ*panCD* induced higher concentrations of these two cytokines (Figures 5H,I).

## Discussion

We report here the assessment of attenuated *M. tuberculosis*Δ*leuD*Δ*panCD* as a suitable and safe model organism for *M. tuberculosis* research, without the need for BSL3 facilities. This strain was originally developed as a TB vaccine candidate (Sampson et al., 2004, 2011) and deletions in the leucine and pantothenate biosynthesis pathways render it safe to work with under BSL2 conditions, since it does not replicate in the absence of exogenous supplementation with leucine and pantothenate. Importantly, we compared *M. tuberculosis*Δ*leuD*Δ*panCD* to *M. tuberculosis* H37Rv with regards to *in vitro* and intra-macrophage growth, response to anti-tuberculosis (TB) agents, genetic background, proteomic response to acid stress and host immune response. Our data supports the suitability of the attenuated strain as a model for TB research.

*M. tuberculosis* H37Rv and *M. tuberculosis* Δ*leuD*Δ*panCD* replicated at similar rates *in vitro* and in murine macrophages; this replication was not influenced by carriage of an additional plasmid. In addition, the auxotrophic nature of *M. tuberculosis* Δ*leuD*Δ*panCD* allows for growth limitation, by removal of either pantothenate (as shown here), or leucine, or both supplements (Sampson et al., 2004). This could provide a useful and tractable stress model as a complement to other commonly used dormancy models (Wayne and Hayes, 1996; Betts et al., 2002; Voskuil et al., 2003, 2004; Leistikow et al., 2010) for investigating latent TB *in vitro*.

A major goal in the field of TB drug development is shortening the course of therapy by identifying new drugs. Being able to do so without the need for a BLS3 facility would greatly decrease cost and increase accessibility to perform drug testing and screening. As proof-of-concept, we compared susceptibility to rifampicin and isoniazid, key first-line TB drugs with different mechanisms of action. Our study shows similar MICs of rifampicin and isoniazid for *M. tuberculosis* Δ*leuD*Δ*panCD* and *M. tuberculosis* H37Rv, although a slightly increased sensitivity was observed for rifampicin for *M. tuberculosis* Δ*leuD*Δ*panCD* compared to *M. tuberculosis* H37Rv. Differences in the observed rifampicin sensitivity for *M. tuberculosis* Δ*leuD*Δ*panCD* could be attributed to the MICs being determined using serial twofold dilutions according to the 1% proportion method; the actual MIC value may therefore be anywhere between the highest drug concentration that allows growth and the last dilution inhibiting growth. The MIC measured for H37Rv may therefore not be 2X the MIC obtained for the attenuated auxotrophic strain, since the precision of the method is considered to be approximately 1 twofold concentration. The observed difference in the MIC for rifampicin between the two strains is therefore within the expected variability of the assay (Kim, 2005; World Health Organisation [WHO], 2018b). However, testing a wider range of anti-TB agents with different mechanisms of action would provide a more comprehensive overview of the inhibition of both strains.

Comprehensive proteomic analysis demonstrated a high level of similarity between *M. tuberculosis*Δ*leuD*Δ*panCD* and *M. tuberculosis* H37Rv when grown under physiological conditions *in vitro*. Only 20 differentially regulated proteins were identified when *M. tuberculosis*Δ*leuD*Δ*panCD* and *M. tuberculosis* H37Rv were grown at pH 6.5, of which 16 were more abundant and four were less abundant in *M. tuberculosis*Δ*leuD*Δ*panCD.* As expected, PanC was absent in *M. tuberculosis*Δ*leuD*Δ*panCD*, which corresponds with the deletion of the *panCD* region from the attenuated *M. tuberculosis* strain (Sampson et al., 2004, 2011).

Potentially also linked to the *panCD* deletion, we observed an increase in relative abundance of a type III pantothenate kinase (CoaX) and a 3-methyl-2-oxobutanoate hydroxymethyltransferase (PanB) that play a role in pantothenate metabolism. PanB upregulation is likely as a result of altered pantothenate metabolism introduced during construction of the *M. tuberculosis*Δ*leuD*Δ*panCD* strain. Supplementation with pantothenate in the culture medium rescues the growth defect incurred by deletion of *panCD* (as previously shown). Pantothenate is phosphorylated during Coenzyme A (CoA) biosynthesis, and CoaX could contribute to the phosphorylation of supplemented pantothenate (Awasthy et al., 2010).

An alkyl hydroperoxide reductase C, AhpC, and an alkyl hydroperoxide reductase, AhpD, were more abundant in *M. tuberculosis*Δ*leuD*Δ*panCD* during growth at pH6.5. AhpD reduces the active site cysteines in AhpC, an NADH-dependent thiol peroxidase, required for the detoxification of peroxides (Hillas et al., 2000; Bryk et al., 2002). Furthermore, a conserved protein (Rv1531) predicted to have peroxiredoxin activity was also more abundant in *M. tuberculosis* Δ*leuD*Δ*panCD*. This indicates that *M. tuberculosis* Δ*leuD*Δ*panCD* may experience increased oxidative and/or nitrosative stress during growth under physiological conditions, as compared to *M. tuberculosis* H37Rv. The transcriptional regulatory protein DevR/DosR was also more abundant in this strain, further supporting a stress response in *M. tuberculosis* Δ*leuD*Δ*panCD* (Supplementary Figure S1). To assess the possibility that the increased DevR/DosR abundance could be caused by excess clumping of the attenuated strain, we performed Ziehl-Neelsen staining of the 2 strains following culture in the presence and absence of Tween 80, as well as at pH 4.5 or pH 6.5 (Supplementary Figure S2). This demonstrated no difference in clumping of the attenuated auxotrophic strain compared to the *M. tuberculosis* H37Rv strain in any of these conditions, indicating that clumping did not influence the DevR/DosR abundance. DevR/DosR is involved in initiating the dormancy response in mycobacteria during exposure to a number of stresses (Sherman et al., 2001; Boon and Dick, 2002; Park et al., 2003; Karakousis et al., 2004; Sharma et al., 2006; Fontán et al., 2008; Kumar, 2008). Taken together, these results indicate that the proteomic profile of *M. tuberculosis*Δ*leuD*Δ*panCD* in normal *in vitro* culture conditions largely recapitulates that of *M. tuberculosis* H37Rv. However, the attenuated strain may be skewed toward a stress response, which should be taken into account during experimental design.

We further probed the stress response of the attenuated strain by comparing proteomic profiles of *M. tuberculosis*Δ*leuD*Δ*panCD and M. tuberculosis* H37Rv following exposure to acid stress. Here, 28 differentially regulated proteins were identified when *M. tuberculosis*Δ*leuD*Δ*panCD and M. tuberculosis* H37Rv were exposed to pH 4.5 for 48 h, of which 24 were more abundant and four were less abundant in *M. tuberculosis*Δ*leuD*Δ*panCD.* As expected, PanC was less abundant in acid-stressed *M. tuberculosis*Δ*leuD*Δ*panCD* when compared to acid-stressed *M. tuberculosis* H37Rv.

We observed several proteomic differences that indicate a possible increased propensity of *M. tuberculosis*Δ*leuD*Δ*panCD* to enter a heightened stress state over *M. tuberculosis* H37Rv. A dormancy-associated translation inhibitor (DATIN, Rv0079) that forms part of the DosR regulon was more abundant in acid-stressed *M. tuberculosis*Δ*leuD*Δ*panCD* (Mishra, 2009; Kumar et al., 2012). *DATIN* gene expression has previously been shown to be upregulated in hypoxic conditions (Voskuil et al., 2004) and to induce pro-inflammatory cytokine expression via interaction with Toll-like receptor 2 (Kumar et al., 2013). A universal stress protein Rv3134c, also a member of the dormancy regulon, was more abundant in acid-stressed *M. tuberculosis*Δ*leuD*Δ*panCD.* This gene is the first member of the *Rv3134c-devR-devS* operon and has been shown to be upregulated during exposure to carbon monoxide (Kumar et al., 2008; Shiloh et al., 2008), nitric oxide (Voskuil et al., 2004) and hypoxic conditions (Sherman et al., 2001). Another member of the dormancy regulon, Rv1738, was also more abundant in acid-stressed *M. tuberculosis*Δ*leuD*Δ*panCD.* The *Rv1738* gene has been shown to be upregulated during exposure to hypoxia (Sherman et al., 2001), carbon monoxide (Kumar et al., 2008), and nitric oxide (Shiloh et al., 2008).

LeuA is involved in leucine biosynthesis and could reflect leucine starvation in the auxotrophic *M. tuberculosis*Δ*leuD*Δ*panCD* strain under acidic conditions. It is highly likely that leucine import could be affected under acidic conditions, placing further stress (in addition to the acidic stress) on *M. tuberculosis*Δ*leuD*Δ*panCD*. Collectively, our proteomic results suggest that *M. tuberculosis*Δ*leuD*Δ*panCD* may exhibit a heightened stress response with associated metabolic changes. Specifically, exposure to an experimentally-induced stress (48 h exposure to pH 4.5) exacerbated this stress response in *M. tuberculosis*Δ*leuD*Δ*panCD*. The *M. tuberculosis*Δ*leuD*Δ*panCD* strain can thus serve as an ideal model to study stress responses in *M. tuberculosis* under BSL2 conditions. Furthermore, exploiting a dual-fluorescent replication reporter and flow cytometry we demonstrated markedly slower *M. tuberculosis*Δ*leuD*Δ*panCD* replication in response to acid stress after 48 h of exposure, compared to *M. tuberculosis* H37Rv. It is thus tempting to speculate that the *M. tuberculosis* Δ*leuD*Δ*panCD* strain will enter a viable, but non-replicating (“dormant” or “persister”) state more readily than *M. tuberculosis* H37Rv when exposed to unfavorable conditions. However, this hypothesis requires further validation.

Another important potential application of attenuated strains is for immunological assays. Although BCG has been widely used for this (Hasso-Agopsowicz et al., 2018; Whittaker et al., 2018), it lacks the RD1 region and as a result it does not secrete many immunogenic proteins. Often cytokine production levels are a major concern with regards to host cells infected by attenuated strains, since many of them have essential immunogenic proteins missing. Here we show that cytokine and chemokine production by PBMCs from individuals infected with *M. tuberculosis* Δ*leuD*Δ*panCD* is not restricted and that several key cytokines (RANTES, GROα, SDF-1 and IL-1β) are produced at comparable levels by PBMCs infected with *M. tuberculosis* Δ*leuD*Δ*panCD* and *M. tuberculosis* H37Rv. While the research question would need to be considered, *M. tuberculosis* Δ*leuD*Δ*panCD* would in many instances be a good representative strain to use as a BSL2-appropriate alternative to *M. tuberculosis* H37Rv for immunological assays.

Interestingly, we observed a higher inflammatory phenotype for PBMCs infected with the attenuated *M. tuberculosis* strain in comparison to the laboratory strain, H37Rv. Specifically, TNFα, GM-CSF, MIP-1α, IL-12p70, and IFNγ were produced at higher levels by PBMCs infected with *M. tuberculosis* Δ*leuD*Δ*panCD* than those infected with *M. tuberculosis* H37Rv. IL-12 plays an important role in anti-tuberculosis cell-mediated immunity, and in addition to IL-18 are regarded as the primary inducers of IFNy production in inflammatory reactions (Trinchieri and Gerosa, 1996; Cooper et al., 1997; Dinarello et al., 1998). Several *M. tuberculosis* strains from different genetic backgrounds have demonstrated differences in the inflammatory response they elicit (Wang et al., 2010; Portevin et al., 2011; van Laarhoven et al., 2013). More specifically, the response of human macrophages to evolutionarily modern strains (bearing the TbD1 deletion, such as Euro-American and Beijing strains) showed a lower cytokine and chemokine production compared to ancestral strains (Portevin et al., 2011). Also, macrophages infected with non-Beijing strains such as Haarlem and LAM, showed heterogeneous cytokine and chemokine production compared to the Beijing strains that tend to induce homogeneously low cytokine and chemokine production. A more recent study has specifically shown that modern Beijing strains show less induction of IL-1ß, IFNγ, and IL-22 *in vitro*, compared to ancient Beijing and Euro-American reactivation strains (van Laarhoven et al., 2013). Despite the highly similar genetic background, the attenuated *M. tuberculosis* Δ*leuD*Δ*panCD* strain elicited higher production of analyzed cytokines, akin to the more “ancient” *M. tuberculosis* lineages. Our proteomic analysis indicated increased production of DATIN in acid-stressed *M. tuberculosis*Δ*leuD*Δ*panCD*, which has previously been implicated in increased proinflammatory cytokine expression (Kumar et al., 2013). It is therefore tempting to speculate that this may contribute to increased inflammatory responses, but this remains to be experimentally determined.

## Conclusion

We provide comprehensive evidence to support the judicious application of *M. tuberculosis* Δ*leuD*Δ*panCD* as a model organism for TB research. The strain recapitulates many characteristics of non-attenuated *M. tuberculosis* H37Rv, and is especially suitable for researchers interested in working with *M. tuberculosis* where access to BSL3 facilities is restricted or unavailable, or where specific instrumentation may not be available in a BSL3 setting. *M. tuberculosis* Δ*leuD*Δ*panCD* may find application in growth-based assays, drug testing, studies of dormancy/persistence, omics analysis (transcriptomics, proteomics and lipidomics), depending on research needs. As with all other models, its suitability should be carefully considered in the context of the research question. However, findings reported here can assist researchers with making an informed choice when using model organisms for tuberculosis research.

## Data Availability

The mass spectrometry proteomics data have been deposited to the ProteomeXchange Consortium (http://proteomecentral.proteomexchange.org) via the PRIDE partner repository with the data set identifier PXD013677. Raw genomic data for this study have been deposited in the European Nucleotide Archive (ENA) under the project accession PRJEB32340.

## Author Contributions

JM and SS conceptualized the experiments and drafted the manuscript. JM performed the *in vitro* and intracellular growth curves, flow cytometry analyses, PBMC assays, and DST testing. TH executed the proteomics analyses. JG and LK contributed to the luminex analyses. AD performed the NGS analyses. JM, TH, AD, JG, LK, and SS contributed to the drafting and revising of the manuscript.

## Conflict of Interest Statement

SS is a named inventor on US Patent US 7,758, 874 B2. The remaining authors declare that the research was conducted in the absence of any commercial or financial relationships that could be construed as a potential conflict of interest.

## Footnotes

1 https://www.r-project.org/

## Supplementary Material

FIGURE S1 Log_2_ LFQ intensities of DevR in *M. tuberculosis*Δ*leuD*Δ*panCD* and *M. tuberculosis* H37Rv during acid stress. No difference in DevR expression was observed between the two strains in acidic conditions (corrected *p*-value of 0.06), based on a Benjamin–Hochberg correction for multiple hypothesis testing. Differences in DevR expression levels are observed between *M. tuberculosis*Δ*leuD*Δ*panCD* and *M. tuberculosis* H37Rv during acid stress, when analyzing protein LFQ intensities without correction for multiple hypothesis testing.

FIGURE S2 Ziehl-Neelsen staining demonstrated no difference in clumping of the attenuated auxotrophic strain compared to the *M. tuberculosis* H37Rv strain in the presence and absence of Tween 80 in media with pH 4.5 or pH 6.5.

TABLE S1 Unique and overlapping variants between *M. tuberculosis* H37Rv and *M. tuberculosis*Δ*leuD*Δ*panCD*.

TABLE S2 All peptides identified in this study.

TABLE S3 All protein groups identified in this study.

TABLE S4 Regulated protein groups in *M. tuberculosis*Δ*leuD*Δ*panCD* versus *M. tuberculosis* H37Rv during 2 days exposure to 7H9 at pH 6.5.

TABLE S5 Regulated protein groups in *M. tuberculosis*Δ*leuD*Δ*panCD* versus *M. tuberculosis* H37Rv during growth in 7H9 at pH 4.5.

DATA SHEET S1 Materials and methods.
