# Supplementary figures and images for: Comprehensive Characterization of the Attenuated Double Auxotroph Mycobacterium tuberculosisΔleuDΔpanCD as an Alternative to H37Rv
Source: Front Microbiol. 2019 Aug 20;10:1922. doi: 10.3389/fmicb.2019.01922 (PMC6710366; doi:10.3389/fmicb.2019.01922)

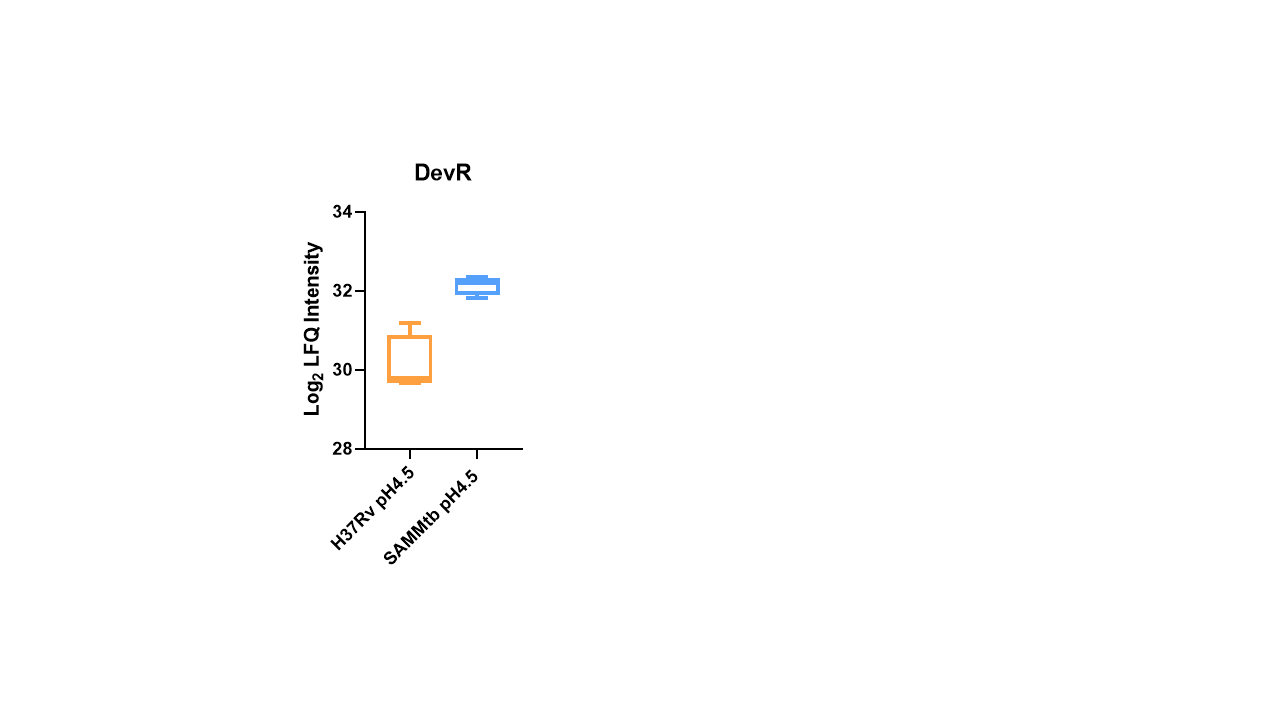

Supplement: FIGURE S1 — Log2 LFQ intensities of DevR in M. tuberculosisΔleuDΔpanCD and M. tuberculosis H37Rv during acid stress. No difference in DevR expression was observed between the two strains in acidic conditions (corrected p-value of 0.06), based on a Benjamin–Hochberg correction for multiple hypothesis testing. Differences in DevR expression levels are observed between M. tuberculosisΔleuDΔpanCD and M. tuberculosis H37Rv during acid stress, when analyzing protein LFQ intensities without correction for multiple hypothesis testing. [file Image_1.TIF]

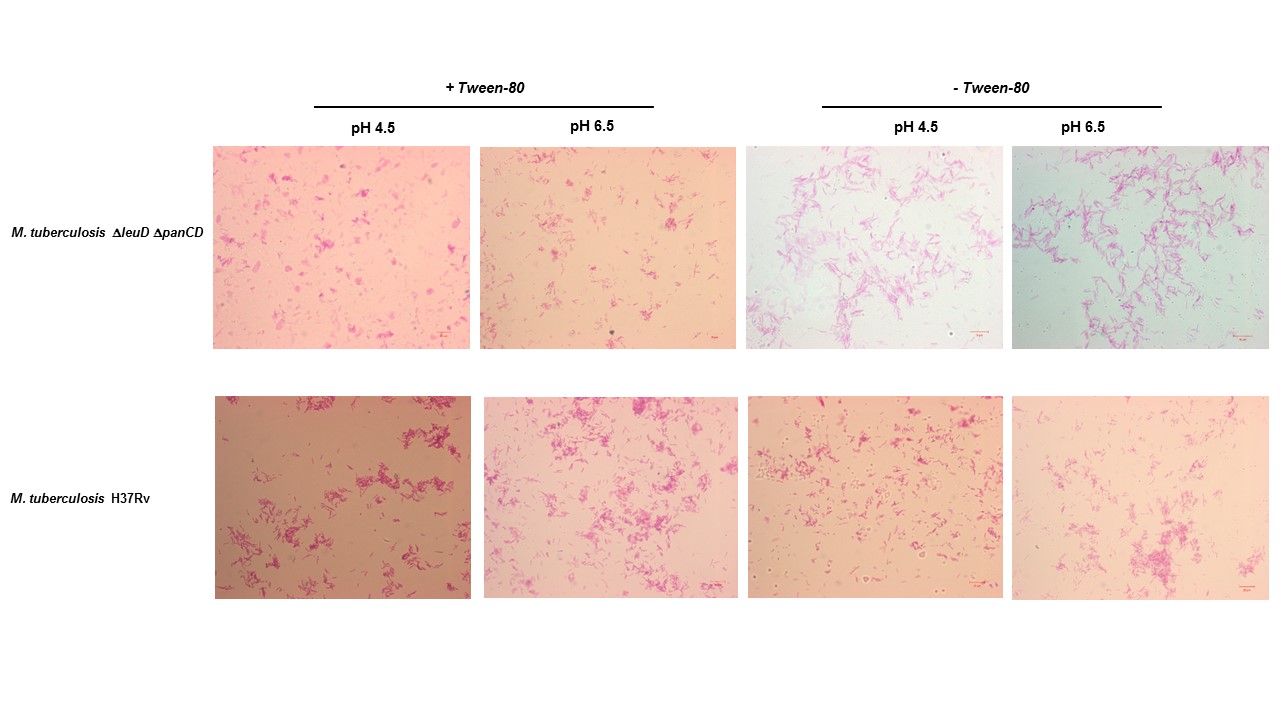

Supplement: FIGURE S2 — Ziehl-Neelsen staining demonstrated no difference in clumping of the attenuated auxotrophic strain compared to the M. tuberculosis H37Rv strain in the presence and absence of Tween 80 in media with pH 4.5 or pH 6.5. [file Image_2.JPEG]
