# Supplementary material for: Comprehensive Characterization of the Attenuated Double Auxotroph Mycobacterium tuberculosisΔleuDΔpanCD as an Alternative to H37Rv
Source: Front Microbiol. 2019 Aug 20;10:1922. doi: 10.3389/fmicb.2019.01922 (PMC6710366; doi:10.3389/fmicb.2019.01922)
Supplement: DATA SHEET S1 — Materials and methods. [file Data_Sheet_1.pdf]

Supplementary Material

1 Materials and Methods

1.1 Protein extractions

Mycobacterial cells from 25 ml culture were suspended in 300 µl lysis buffer consisting of 8 M urea, 50 mM triethylammonium bicarbonate (TEAB), 5 mM tris (2-carboxyethyl)phosphine) (TCEP) and containing EDTA-free protease inhibitor cocktail (Roche, Basel Switzerland). Cells were disrupted by mechanical bead-beating, using glass beads (425-600 µm, Sigma-Aldrich) and a Ribolyser (Bio101 SAVANT, Vista, California, USA). Lysis steps consisted of 8 cycles of 20 s each, at a speed of 4.0 $m.s^{-1}$. Samples were cooled on ice for 1 minute between each lysis step. Whole-cell lysates were centrifuged (12 000 g, 10 minutes), the supernatants transferred to a new microfuge tube and centrifuged once more (12 000 g, 10 minutes) to remove all cellular debris. Whole-cell lysates were filter-sterilized using a 0.22 µm PVDF membrane before removal from the BSL3 facility. Protein concentration was determined using a Bradford method (Bio-Rad, California, USA).

1.2 Filter-aided sample preparation and trypsin digestion of protein samples

Proteomic samples were processed using a modified version of the filter-aided sample preparation (FASP)(Wiśniewski et al., 2009). Briefly, 40 µg protein from each replicate was reduced with 5 mM TCEP at room temperature for 1 hour and subsequently alkylated with 5.5 mM iodoacetamide for 1 hour in the dark. Reduced and alkylated protein samples were transferred to a 0.5 ml Amicon ultra 30 kDa cut-off spin filter (Millipore, Massachusetts, USA) and centrifuged at 12 000 g for 15 minutes. Four hundred microlitres UB buffer (8 M urea in 50 mM TEAB) was added to each filter, and the samples were centrifuged (12 000 g, 15 minutes). This process was repeated twice for a total of three UB washes. Four hundred microlitres 50 mM TEAB was added to each filter, and the samples were centrifuged (12 000 g, 15 minutes). This was repeated twice for a total of three TEAB washes. Proteins were on-filter digested for 24 h at 37 °C, using a 1:50 ratio of trypsin to protein. The filter unit was placed in a new collection tube after digestion, and the peptides were obtained in the flow-through by centrifugation (12 000 g, 15 minutes). Peptides were eluted once more from the filter unit by the addition of 250 µl 100mM NaCl and further centrifugation (12 000 g, 15 minutes). Peptide samples were desalted using in-house packed STAGE-tips, prepared with Empore™ C18 SPE disks (Sigma-Aldrich), and dried before storage at -20 °C.

1.3 Mass spectrometry

A total of 1 µg peptide mixture from each sample was analysed, independently, on an Orbitrap Fusion Tribrid mass spectrometer (Thermo Fisher Scientific), connected to a *Thermo* Scientific *UltiMate 3000 RSLCnano System (*Thermo Fisher Scientific*)*. Peptide separation was performed on a PepMap 300 C18 LC pre-column (300 µm ID x 5 mm, 5 µm, 300 Å), followed by separation on an analytical column (75 µm ID x 350 mm) packed with C18 Aeris Peptide 3.6 µm beads (Phenomenex 04A-4507), at a flow rate of 300 nL/min. Solvent A was 2% acetonitrile in 0.1% formic acid, and solvent B was 100% acetonitrile in 0.1% formic acid. The gradient used was as follows: solvent B was maintained at 2% for 10 minutes, followed by an increase from 2 to 10% B in 5 minutes, 10-35% B in 70 minutes, 35-50% B in 10 minutes, 50-80% B in 0.1 minutes, and maintained at 80% B for 10 minutes. The Orbitrap

Fusion was operated in positive ion data-dependent mode for Orbitrap-MS and Orbitrap-MS2 data acquisition. Data were acquired using the Xcalibur software package. The precursor ion scan (full scan) was performed in the Orbitrap in the range of 350-1500 m/z with a nominal resolution of 120 000. The number of accumulated ions was set to 4 x 10^5. Ion filtering for Orbitrap-MS2 data acquisition was performed using the quadrupole with a transmission window of 1.5 m/z. The most intense ions above an intensity threshold of 5×10^3 were selected for high-energy collisional dissociation (HCD). An HCD normalized collision energy of 32.5% was applied to the most intense ions, and fragment ions were analysed in the Orbitrap at a resolution of 30 000. The number of Orbitrap-MS2 events between full scans was determined on-the-fly to maintain a 3s fixed duty cycle. Dynamic exclusion of ions within a ±10 p.p.m. m/z window was implemented using a 30 s exclusion duration. An electrospray voltage of 2.0 kV and capillary temperature of 280 °C, with no sheath and auxiliary gas flow, was used. The automatic gain control (AGC) settings were 4×10^5 ions with a maximum ion accumulation time of 50 ms for Oribtrap-MS, and 5×10^4 ions with a maximum ion accumulation time of 50 ms for Orbitrap-MS2 scans, respectively. Ions with <2+ or undetermined charge state were excluded from MS2 selection.

1.4 Data analysis

For data analyses of the multiplex bead array assay the distribution of the raw data was assessed with Quantile-Quantile (QQ) plots against various distributions using the QualityTools package (https://cran.r-project.org/web/packages/qualityTools/citation.html) in Rstudio version 3.4.0. The raw data followed a log-normal distribution, and therefore we applied a log transformation which resulted in normally distributed residuals as tested with QQ plots and histograms. Missing values that fell below the detection limit of the standard curve for each cytokine, across patients were imputed with the lower quantile values based on a truncated normal distribution in Perseus (Tyanova et al., 2016). We inspected the imputation with QQ plots and verified that the data remains normally distributed. The residuals comprising of transformed and imputed data was used to test for equal variances using the Levene's test, all values were greater than 0.05 indicating that data between samples were of equal variance. As the assumptions for parametric testing were met, we used a one-way analysis of variance (ANOVA) hypothesis test with a TukeyHSD post hoc test, with an adjusted p value cut off of 0.05, to evaluate the difference between means using Graphpad Prism version 5 (GraphPad Software Inc., CA).

All tandem mass spectra were analysed using MaxQuant 1.5.5.1 (Cox and Mann, 2008), and searched against a customized *M. tuberculosis* proteome database. Custom database construction was performed as previously described (Heunis et al., 2017). Briefly, proteins containing single amino acid variants, derived from WGS analysis, were concatenated to the *M. tuberculosis* reference proteome (downloaded from Uniprot on 26 September 2017, containing 3993 entries). The custom *M. tuberculosis* database contained 4012 entries. Peak list generation was performed within MaxQuant and searches were performed using default parameters in MaxQuant. An initial search precursor mass tolerance of 20 ppm and a fragment mass tolerance of 0.5 Da was employed. The enzyme specificity was set to consider fully tryptic peptides, and two missed cleavages were allowed. Carbamidomethylation of cysteines was set as fixed modification, and oxidation of methionine and N-terminal acetylation was allowed as variable modifications. Proteins were considered confidently identified when they contained at least two unique tryptic peptides, employing a protein and peptide false discovery rate (FDR) of less than 1% in MaxQuant. Proteins that contained similar peptides and that could not be differentiated based on tandem mass spectrometry analysis alone were grouped to satisfy the principles of parsimony. Reverse hits, contaminants and proteins only identified by site were

removed before downstream statistical and bioinformatics analysis. A label-free quantification strategy was employed using the MaxLFQ algorithm (Cox et al., 2014) within MaxQuant. Assigned LFQ values were used for statistical analysis in Perseus 1.6.0.7 (Tyanova et al., 2016). Data were transformed (log 2) and filtered to contain at least three valid LFQ values in one group for pair-wise comparisons. Missing values were imputed using random numbers drawn from a normal distribution that simulates signals from low abundance proteins. Welch's t-tests were performed, and p-values were corrected for multiple hypothesis testing using the Benjamini-Hochberg FDR method.

1.5 References

Cox, J., Hein, M.Y., Luber, C.A., Paron, I., Nagaraj, N., and Mann, M. (2014). Accurate proteome-wide label-free quantification by delayed normalization and maximal peptide ratio extraction, termed MaxLFQ. *Mol. Cell. Proteom.* 13(9)**,** 2513-2526. doi: 10.1074/mcp.M113.031591.

Cox, J., and Mann, M. (2008). MaxQuant enables high peptide identification rates, individualized p.p.b.-range mass accuracies and proteome-wide protein quantification. *Nat. Biotechnol.* 26**,** 1367. doi: 10.1038/nbt.1511

Heunis, T., Dippenaar, A., Warren, R.M., van Helden, P.D., van der Merwe, R.G., Gey van Pittius, N.C., et al. (2017). Proteogenomic investigation of strain variation in clinical *Mycobacterium tuberculosis* isolates. *J. Proteom. Res.* 16(10)**,** 3841-3851. doi: 10.1021/acs.jproteome.7b00483.

Tyanova, S., Temu, T., Sinitcyn, P., Carlson, A., Hein, M.Y., Geiger, T., et al. (2016). The Perseus computational platform for comprehensive analysis of (prote)omics data. *Nat. Meth.* 13**,** 731. doi: 10.1038/nmeth.3901

Wiśniewski, J.R., Zougman, A., Nagaraj, N., and Mann, M. (2009). Universal sample preparation method for proteome analysis. *Nat. Meth.* 6**,** 359. doi: 10.1038/nmeth.1322
